# Arginine Vasopressin Deficiency Heralding Rosai-Dorfman Disease With Neurological Manifestations

**DOI:** 10.1210/jcemcr/luae206

**Published:** 2024-11-07

**Authors:** David Felske, Jacob Gabbay, Brittney Boles, Matthew P Gilbert

**Affiliations:** Division of Endocrinology & Diabetes, The University of Vermont Larner College of Medicine, Burlington, VT 05405, USA; Department of Medicine, The University of Vermont Larner College of Medicine, Burlington, VT 05405, USA; Department of Pathology & Laboratory Medicine, The University of Vermont Larner College of Medicine, Burlington, VT 05405, USA; Division of Endocrinology & Diabetes, The University of Vermont Larner College of Medicine, Burlington, VT 05405, USA

**Keywords:** Rosai-Dorfman disease, central diabetes insipidus, arginine vasopressin deficiency, non–Langerhans cell histiocytosis

## Abstract

Rosai-Dorfman disease (RDD) is a rare heterogeneous disorder of non–Langerhans cell histiocytosis. The patient is a 24-year-old woman who presented with a 1-month history of polydipsia, polyuria, and 25-lb (11.3-kg) weight loss over 6 months and was found to have significantly elevated 24-hour urine volume (8.4 L). Prior to completion of the work-up for her presenting symptoms, she returned with a new complaint of disabling back pain and bilateral lower-extremity numbness with weakness refractory to conservative treatment. Magnetic resonance imaging (MRI) showed a prominent T2 to T4 stenosis from a soft tissue mass. Due to progressive pain and accelerating neurological symptoms, she was admitted for surgical debulking and biopsy. In the 10 hours she was unable to drink fluids surrounding her procedure, her serum sodium climbed to 160 mmol/L (reference interval, 137-145 mEq/L; [137-145 mmol/L]). Urine testing and desmopressin challenge revealed arginine vasopressin deficiency (AVP-D), formerly known as central diabetes insipidus. Pituitary MRI showed a mildly enlarged pituitary gland with loss of normal posterior pituitary signal supporting the diagnosis. Epidural mass pathology showed predominant histiocytes indicating RDD. This case highlights the diverse clinical manifestations of RDD and is an unusual instance of RDD linked with AVP-D and neurological involvement.

## Introduction

Histiocytic disorders are classified broadly into 5 categories based on clinical histology, phenotype, molecular alterations, clinical, and imaging characteristics [1]. These categories include Langerhans related, cutaneous and mucocutaneous, malignant histiocytosis, Rosai-Dorfman disease (RDD), hemophagocytic lymphohistiocytosis and macrophage activation syndrome [1]. RDD, also known as sinus histiocytosis with massive lymphadenopathy, is a rare disorder of non–Langerhans cell histiocytosis with an annual incidence of fewer than 5 per million individuals. The disease is seen more often in children and young adults; however, incidence reports have occurred up to age 74 years [2]. It is known for its diverse clinical manifestations and difficulty in diagnosis [3]. The disease most commonly manifests with cervical lymphadenopathy, but areas of involvement can also include the skin, central nervous system, orbit, upper respiratory tract, bone, and retroperitoneum [3]. While central nervous system involvement is a known occurrence, it is present in less than 5% of cases [4] with only a fraction of those involving the suprasellar region [5]. After careful literature review, the disease has never been heralded by arginine vasopressin deficiency (AVP-D), also called diabetes insipidus, with spinal involvement. In this case report, we describe a patient who presents with a previously undocumented complication of a rare disorder.

## Case Presentation

The patient is a 24-year-old woman who presented to her primary care physician with a 1-month history of polydipsia, polyuria, headache, blurry vision, and 25-lb (11.3-kg) weight loss over 6 months. She had a history of generalized anxiety disorder, depression, and chronic interstitial cystitis. Her medication list included only cyclobenzaprine and lorazepam as needed. Initial laboratory work was most notable for sodium of 145 mEq/L (145 mmol/L) (reference interval, 137-145 mEq/L; [137-145 mmol/L]), with glycated hemoglobin A_1c_ 5.1% [51 g/L] (reference interval, <5.7% [<57 g/L]). Twenty-four–hour urine studies were performed that showed a urine volume of 8400 mL, with urine sodium 202 mEq/24 hour (202 mmol/24 hour) (40-220 mEq/24 hour [40-220 mmol/24 hour]) and urine osmolality 88 mOsm/kg (88 mmol/kg) (reference interval, 301-1090 mOsm/kg H_2_O [301-1090 mmol/kg]). It was recommended that she undergo a water deprivation test to differentiate between AVP-D and primary polydipsia, but she did not pursue further testing.

**Figure 1.**
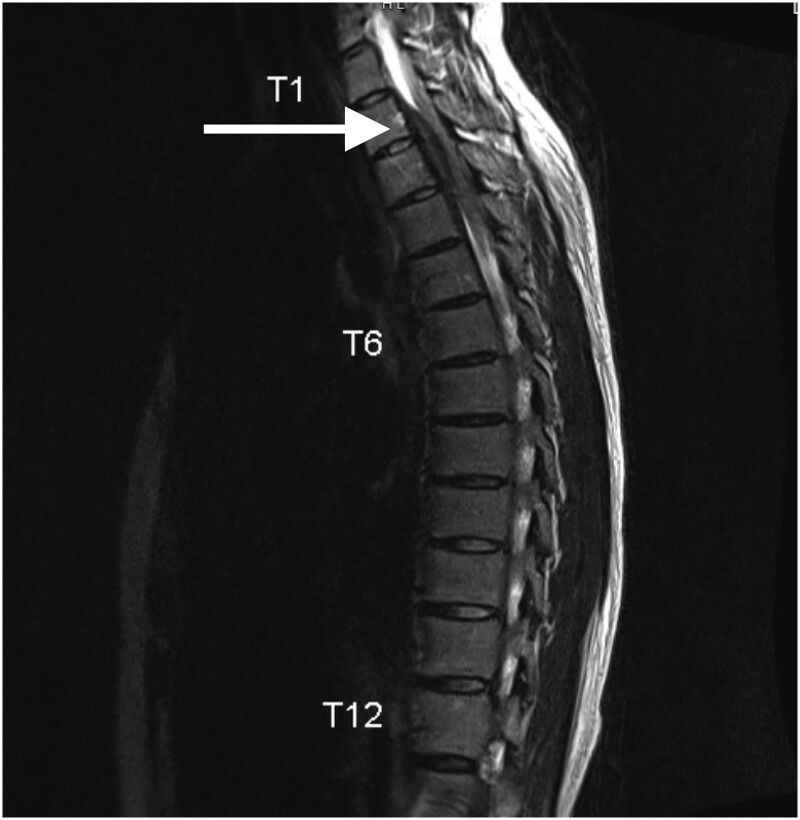
Initial magnetic resonance imaging of the thoracic spine showing prominent T2 to T4 stenosis from unknown soft tissue mass, sagittal view.

She returned to her primary care provider approximately 2 months later for worsening, atraumatic, thoracic back pain that was initially thought to be musculoskeletal in nature. Her pain was only partially relieved by conservative care including acetaminophen, nonsteroid anti-inflammatory agents, muscle relaxants, and alternating ice and heat. Lumbar x-ray showed a mild rightward curvature with spondylosis, but no acute disease. The pain continued to worsen to the point where she had difficulty with her activities of daily living, and further imaging was recommended. Magnetic resonance imaging (MRI) of her thoracic spine revealed a prominent stenosis between T2 and T4 with a low signal area anterior to the cord (Fig. 1). She was referred to neurosurgery for further evaluation. While awaiting follow-up, she developed progressive weakness and numbness resulting in a fall, which prompted her presentation to the emergency department. Computed tomography scan of the thoracic spine redemonstrated a stenotic lesion that had not changed in size. Due to progressive symptoms suggestive of spinal cord compression, she was admitted to the hospital and solids/liquids were held prior to emergent spinal decompression. She had an uneventful surgical debulking with no complications, but she remained intubated for approximately 36 hours.

**Figure 2.**
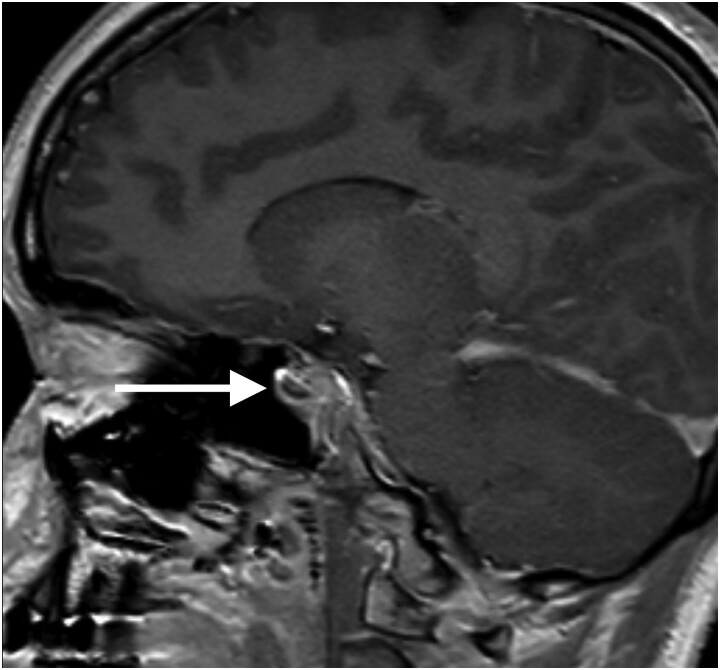
Magnetic resonance imaging of the pituitary showing mild enlargement with loss of normal posterior signal.

Over the 10 hours solids/fluids were held surrounding her procedure, the patient’s sodium increased from 143 to 160 mEq/L (143-160 mmol/L) (reference interval, 137-145 mEq/L; [137-145 mmol/L]). Urine studies showed osmolality of 109 mOsm/kg H_2_O (109 mmol/kg) (reference interval, 301-1090 mOsm/kg H_2_O [301-1090 mmol/kg]). She was given a 5% dextrose in water (D5W) bolus and trialed on 0.01 mcg of intravenous desmopressin (dDAVP) for presumed AVP-D. Urine osmoles increased from 109 to 689 mOsm/kg (109 to 689 mmol/kg) (reference interval, 301-1090 mOsm/kg H_2_O [301-1090 mmol/kg]) following treatment with dDAVP. Pituitary MRI supported a diagnosis of AVP-D with mild enlargement of the pituitary gland and loss of the normal signal of the posterior pituitary (Fig. 2).

## Diagnostic Assessment

The spinal lesion was nonspecific on MRI, and differential considerations from an imaging perspective included primarily metastasis vs infection.

**Figure 3.**
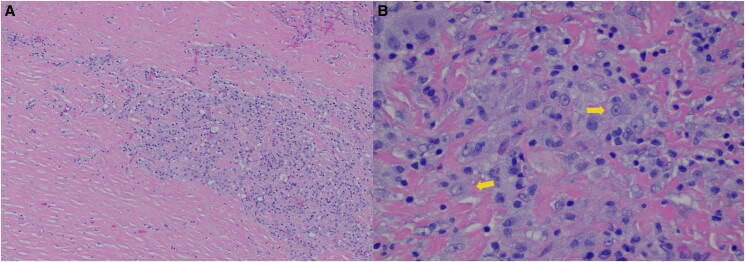
A, Left shows poorly defined borders of inflammatory cells in a background of hyalinized stroma. B, Right shows an abundance of histiocytes (arrow).

**Figure 4.**
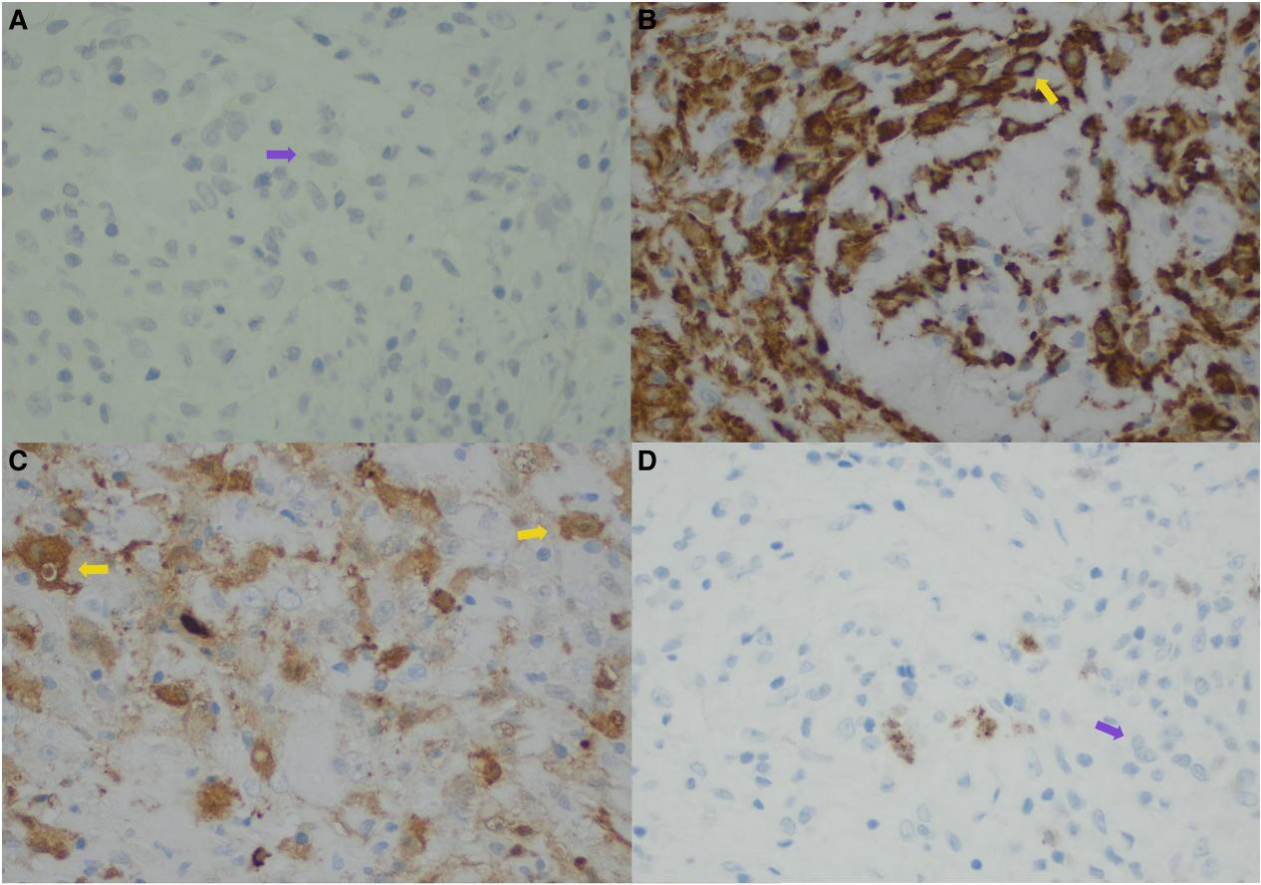
B, CD163 is positive in histiocytes (yellow arrow). C, S100 is positive in Rosai-Dorfman histiocytes (yellow arrow). A, CD1a and D, langerin are negative in the histiocytes (purple arrow).

The epidural biopsy showed a lesion with sheets of pleomorphic cells with a moderate amount of cytoplasm and poorly defined borders. Immunohistochemical stains confirmed the lesion was predominantly composed of histiocytes with a background of lymphocytes (Fig. 3). Additional immunohistochemical stains were performed to further characterize the process, showing the histiocytes were positive for S100 and negative for Langerin and CD1a (Fig. 4) This is consistent with RDD. Notably, emperipolesis, which is a unique feature of RDD, is inconspicuous in this case. Emperipolesis describes the phagocytosis of lymphocytes or plasma cells within the cytoplasm of histiocytes. This has been reported with the extranodal form of the disease as well as admixed foamy histiocytes, which were also seen in this specimen, and does not contradict the immunohistochemical characterization. In pursuit of possible skin involvement, a skin biopsy was taken that showed only scant terminal inflammation.

## Treatment

For treatment of AVP-D, the patient was started on dDAVP with the goal of symptom improvement, decreasing nocturia, and making daytime polyuria tolerable. Care was taken to ensure she was on the minimal effective dose, so she was slowly up titrated over 5 days and eventually stabilized on 0.05-mg tablets in the morning and at lunchtime with a 0.01-mg nasal spray at night. RDD was treated with the oral administration of prednisone 60 mg daily and methotrexate 25 mg weekly.

## Outcome and Follow-up

After 5 months on prednisone and methotrexate, the patient’s symptoms failed to improve with relatively stable imaging. She was offered radiation therapy, but she declined. As an alternative, she was started on 6 mercaptopurine 50 mg/m^2^/day with methotrexate, and her prednisone was discontinued. Despite the new regimen, her symptoms persisted and there was MRI evidence of increasing burden of disease in her spine. This prompted additional staining of original biopsy with cyclin D1. This came back positive, and she was started on cobimetinib (a mitogen-activated extracellular kinase pathway inhibitor). Since that time, she has had substantial symptom improvement. Recent MRI of the pituitary showed interval decrease in the size of the pituitary gland and her morphology returned to normal. Repeat MRI imaging of the spine shows interval resolution of the soft tissue lesions, with no new lesions reported. The patient has continued stable doses of dDAVP after 1 year of follow up, which may suggest permanent AVP-D.

## Discussion

One of the unique features in this case is the initial clinic presentation of AVP-D, which heralded the later development of cauda equina syndrome requiring emergent debulking of the spinal mass. AVP-D is classically associated with other histiocytic disorders such as Langerhans cell histiocytosis and Erdheim-Chester disease but has rarely been related to RDD without involvement of an intracranial mass. Following literature review, there are some case reports of AVP-D and RDD; however, most presented with a suprasellar or sellar mass [6]. There was one case of a 44-year-old woman with xanthelasma-like cutaneous lesions on the facial area and neck with a diagnosis of RDD who presented concomitantly with AVP-D, however no brain MRI imaging was reported [7].

One previous case report noted a 45-year-old woman who presented with pyrexia, headaches, and hyponatremia who was found to have a suprasellar tumor arising from the posterior pituitary tumor. One year later, she developed a T2 intradural enhancing mass that histological examination was consistent with RDD [8]. Both the suprasellar and spinal tumors were removed; however, 3 years later, she developed AVP-D. Given the time delay, AVP-D may have developed due to a histiocytic infiltrative process independent of a compression effect from a brain tumor. In conjunction with our case, this may suggest that RDD may have a targeted effect on the posterior pituitary gland and can lead to the development of AVP-D.

Consensus recommendations for the diagnosis and clinical management of RDD were released in June 2018 [2]. There is currently no uniform approach to RDD, and treatment is individualized. Abla et al [2] summarize a variety of strategic approaches and have proposed a management algorithm. In this patient's case, surgical debulking was performed given symptoms and imaging concerning for spinal cord compression, and the tissue sample was used to establish the diagnosis. AVP-D was diagnosed through a significant increase in low urine osmolality following dDAVP administration. Newer diagnostic methods include measuring copeptin, which mirrors arginine vasopressin while also being stable and easily measured. Measurement of copeptin following osmotic stimulation by hypertonic saline solution has been showed to have greater diagnostic accuracy than the water deprivation test in patients with hypotonic polyuria [9].

Given the residual spinal tumor in the lumbar region, systemic therapy including high-dose corticosteroids and chemotherapy was performed. Imaging revealed that the spinal tumor was unresponsive to systemic treatment, and she was switched to targeted therapy with cobimetinib following repeat staining. Follow-up imaging of the pituitary gland following treatment showed interval shrinking in the size of the pituitary gland, which may suggest a response to systemic targeted treatment.

In conclusion, our case report is a rare case of AVP-D and RDD occurring with the development of severe neurological symptoms from a multifocal spinal mass. This case illustrates the importance of appropriate work-up of polyuria including water deprivation, and clinicians should be advised to consider other neurological involvements in the isolated development of AVP-D.

## Learning Points

- Histiocytosis (in this case RDD) is a potential cause of spontaneous AVP-D.
- The differential diagnosis of vasopressin deficiency is broad.
- Alert other clinicians when evaluating a patient for AVP-D.
- dDAVP titration is challenging; the goal is using the lowest effective dose.
- RDD is a heterogeneous disease with possible multisystem involvement.

## Data Availability

Data sharing is not applicable to this article as no data sets were generated or analyzed during this study.

## Abbreviations

AVP-D: arginine vasopressin deficiency
dDAVP: desmopressin
MRI: magnetic resonance imaging
RDD: Rosai-Dorfman disease
